# Involvement of Hookworm Co-Infection in the Pathogenesis and Progression of Podoconiosis: Possible Immunological Mechanism

**DOI:** 10.3390/tropicalmed3020037

**Published:** 2018-03-26

**Authors:** Damilare O. Famakinde, Adedotun A. Adenusi

**Affiliations:** Department of Medical Microbiology and Parasitology, College of Medicine, University of Lagos, Idi-Araba, Surulere P.M.B 12003, Lagos 100254, Nigeria; aaadenusi@yahoo.com

**Keywords:** podoconiosis, hookworm, iron deficiency anemia, co-infection, immunopathogenesis, inflammation, fibrosis

## Abstract

Podoconiosis is an endemic, non-infectious, geochemical and non-filarial inflammatory cause of tropical elephantiasis. The immunology of podoconiosis is not yet expressly understood. In spite of this, co-infection and co-morbidity with the infectious, soil-transmitted hookworm disease that causes iron deficiency anemia has been found to be predominant among affected individuals living in co-endemic settings, thus creating a more complex immunological interplay that still has not been investigated. Although deworming and iron-rich nutrient supplementation have been suggested in podoconiosis patients living under resource-poor conditions, and it is thought that hookworm infection may help to suppress inflammatory responses, the undisputed link that exists between a non-infectious and an infectious disease may create a scenario whereby during a co-infection, treatment of one exacerbates the other disease condition or is dampened by the debilitation caused by the other. In this paper, we elaborate on the immunopathogenesis of podoconiosis and examine the possible immunological dynamics of hookworm co-infection in the immunopathology of podoconiosis, with a view toward improved management of the disease that will facilitate its feasible elimination.

## 1. Introduction

Podoconiosis is an endemic, non-infectious, geochemical and inflammatory disease caused by chronic barefoot contact with mineral particles present in certain volcanic clay soils, resulting in bilateral lymphedema and disfigurement of the lower legs [1]. The disease has long been identified as a distinct entity [1] and in February 2011, the World Health Organization (WHO) listed it as one of the 20 most neglected tropical diseases (NTD) [2]. It ranks second only to lymphatic filariasis as the most common cause of tropical lymphedema, which is otherwise known as elephantiasis [3]. Furthermore, it places enormous economic and psychosocial burdens on affected populations [4,5,6,7]. Estimates suggest that 4 million people are affected globally, who are mainly in tropical Africa but also in Central and South America and Southeast Asia [8]. The global distribution of podoconiosis has been under-reported. In recent literature, the disease has been reported in 32 countries worldwide, some of which are suspected or known to be endemic [8]. It is believed that Ethiopia, with around 1.5 million cases (more than 25% of the global total) [9], bears the highest global burden of podoconiosis. It is estimated that 24% of the overall land surface of the country area, on which 43.8% of the national population lives [10], is covered by the irritant red clay [11].

Agrarian individuals, who walk and work barefoot in the fields of red clay soils, and individuals living in houses with uncovered (mud or earth) floors are at risk [7,12]. The causative irritant microparticles, which are notably crystalline silica (*c*SiO_2_), alumino-silicate (Al_2_SiO_5_), stacked kaolinite (Al_2_Si_2_O_2_[OH]_4_), iron oxide (Fe_2_O_3_) and zirconium (Zr), among others [13,14], are absorbed into the skin and their progressive passage into the lymphatics causes damage to the endothelium of the lymph channels and vessels as well as to the valves of lymph vessels and lymph nodes [15]. Itching and splaying of the forefoot, increased skin markings, painful acute lymphangioadenitis, skin nodules and papillomata formation, hyperkeratosis, rigid toes, the fusion of the interdigital spaces and ankylosis of the interphalangeal or ankle joints, are pathognomonic for podoconiosis [1]. It is important to note that toxicity of the irritant particles is concentration dependent [16] and human susceptibility is subject to genetic influence [17]. For easy staging of disease severity, Tekola et al. [18] delineated 5 clinical stages in podoconiosis, although it is important to underline that these stages do not necessarily represent the disease process.

The infectious but neglected hookworm diseases, necatoriasis and ancylostomiasis, are also widely found in the tropics with their highest prevalence occurring in Asia and sub-Saharan Africa [19,20]. Infections occur through percutaneous penetration of the soil-dwelling, filariform larvae (L3) of the respective causative parasites, *Necator americanus* and *Ancylostoma duodenale* (also the zoonotic *A. ceylanicum*), although this can also occur through the fecal–oral route in *A. duodenale* [19,21,22]. The adult parasites inhabit the small intestine of humans and may cause severe iron deficiency anemia (IDA) in infected individuals, especially those with heavy worm burdens [19,23,24]. Although the degree of hookworm-induced IDA depends on the infecting worm species and anemia may occur even with a lighter worm burden [19], it is generally believed that in light and moderate hookworm infections, anemia results primarily due to insufficient iron intake worsened by the presence of the worms. However, in heavy infections, anemia ensues even when adequate dietary intake is maintained [24]. Globally, 5.1 billion people are thought to be at risk of acquiring hookworm infection, of which approximately 500 million people are currently affected [25]. According to the recent WHO global health estimates, hookworm diseases account for over 1.7 million disability-adjusted life years (DALYs) [26].

Co-infections are not uncommon among individuals living in co-endemic areas. The undisputed complex link that exists between non-infectious and infectious diseases may create a scenario whereby during a co-infection, treatment of one exacerbates the other disease condition or is dampened by the debilitation caused by the other. Higher hookworm infection rates have been reported among podoconiosis patients [27], especially during the early clinical stages. Those with complicated and advanced podoconiosis would be prevented from working on farms due to resulting incapacitation, consequently resulting in them having less frequent contact with the soil and thus, the soil-transmitted helminths [27,28]. According to Taye et al. [27], hookworm infections were found in 40.9% of podoconiosis patients but in only 27.5% of the human controls living in the same endemic area. This may attribute to the higher percentage (33%) of anemia cases found among podoconiosis patients, which reaches well above the 15.25% cases of anemia found in the endemic controls [27]. Moreover, the mean hemoglobin level was 13.5 g/dL in podoconiosis-hookworm patients compared with the 14.5 g/dL observed among podoconiosis-unaffected but hookworm-infected individuals [27]. According to WHO [29], anemia occurs at a hemoglobin concentration below 13 g/dL in men over 15 years of age, below 12 g/dL in non-pregnant women over 15 years of age, and below 11 g/dL in pregnant women. However, slight age- and race-related variations exist [30].

The geographical and occupational overlaps between both podoconiosis and hookworm infection are considered as key predisposing factors for co-infection [28]. Nevertheless, it is yet unknown how this co-infection may influence the pathogenesis of podoconiosis, exacerbate its pathology or impair the effectiveness of the current available treatments, such as foot hygiene, foot elevation, compressive bandaging and surgical nodulectomy. Interestingly, deliberate light infection with viable human hookworm (*N. americanus*) is emerging as a possible therapy for some human inflammatory diseases, although this has not yet been validated [31,32]. Elimination of podoconiosis is easible [33], although this will further be hastened by a good understanding of the various spectra of the disease pathogenesis that may provide insights toward improved disease management. This review elucidates the possible involvement of hookworm co-infection in the immunopathogenesis and progression of human podoconiosis.

## 2. Immunology of the Pathogenesis of Podoconiosis

The immunopathogenesis of podoconiosis is complex and involves a plethora of immune factors and cells. Macrophages are among the first major sets of innate cells that mount a defense against the invading foreign microparticles. Based on their activation profiles, the macrophages may be categorized as classically activated (M1) and alternatively activated (M2) macrophage phenotypes. The M1 macrophages, also considered as proinflammatory macrophages, are polarized by tumor necrosis factor-α (TNF-α), interferon-γ (IFN-γ) and lipopolysaccharide (LPS). As a result, they produce proinflammatory cytokines, such as interleukin-1β (IL-1β), IL-8, IL-6, IL-12 and TNF-α. In contrast, the profibrotic cytokines IL-4 and IL-13 induce polarization of the M2 macrophages, characterized by the production of tissue-repairing IL-10 [34,35]. The prolonged delay in podoconiosis development despite constant exposure to the irritant microparticles may be attributed to the sequestration of the microparticles by the M2 macrophages, which are relatively insensitive to inflammatory stimuli but express abundant levels of scavenger receptors [36]. At a certain threshold beyond which the M2 macrophages are overloaded and are unable to accommodate more particles, most free particles become engulfed by the inflammatory M1 macrophages [36].

Phagocytosis of mineral particles by the M1 macrophages stimulates the macrophages to release reactive oxygen species (ROS) and inducible nitric oxide synthase (iNOS). Simultaneously, they release nuclear factor-kappa B (NF-kB) and activator protein-1 (AP-1), which trigger the production and subsequent release of inflammatory cytokines. These cytokines, which are majorly TNF-α, IL-1 and IL-6, combine with proteases (e.g., matrix metalloproteinases or MMPs), arachidonic acid/eicosanoid metabolites (leukotriene-B4, prostaglandin E2 or PGE2), mesenchymal cell growth-promoting factors and other mediators to invoke an inflammatory response [37], such as endolymphangitis. Unsuccessful particle clearance may cause apoptosis of the particle-containing macrophages, leading to release of the particles, which are then re-engulfed by other M1 macrophages. This induces a cycle of injury accompanied by the infiltration of lymphocytes, mast cells, plasma cells, neutrophils as well as inflammatory cytokines, chemokines, macrophage inflammatory proteins (MIPs) and monocyte chemoattractant proteins into the injured area, resulting in further inflammatory changes [36,37], such as the painful acute lymphangioadenitis.

Fibrosis ensues as a result of dysregulated and prolonged wound healing or connective tissue repair in response to recurring lymphatic tissue microinjuries [38]. During the aberrant wound healing and fibrosis development, fibroblasts hyperproliferate at the site of injury, acquire a profibrotic phenotype that is resistant to apoptosis and differentiate into contractile myofibroblasts that perpetuate the fibrotic process [38]. Fibroblasts are the key cells responsible for the synthesis and deposition of extracellular matrix (ECM) [38]. Excessive deposition of ECM components, such as collagen and fibronectin, is a hallmark of the fibrotic repair process and irreversibly remodels the lymphatic tissue structure [38]. This causes subendothelial edema, increased thickening of lymphatic walls (causing progressive reduction of the lymphatic lumen until its complete blockage), simultaneous impairment of lymph flow, lymph stasis and consequently, lymphedema [39,40]. In podoconiosis, the rims of collagen around dilated blood vessels were found to be collagen IV-positive and the vascular systems were positive for cluster of differentiation 31 (CD31) [41]. In advanced stages, the accumulation of adipocytes, keratinocytes and fibroblasts transforms the initially soft swollen tissue into a hard fibrotic mass and a stiff, thickened hyperkeratotic skin [42].

Both innate and adaptive immune cells modulate inflammation and fibrogenesis via different mechanisms. The adaptive immune response is activated through antigen presentation and antigenic stimulation by the macrophages and other antigen-presenting cells. Studies conducted on affected individuals have demonstrated that podoconiosis is adaptively a T-cell-mediated inflammatory condition [17,41], although 30% of the lymphocytic infiltrates in podoconiosis nodular tissue consists of B cells [41]. CD4^+^ T cells have important role in the pathogenesis and progression of fibrosis, depending on the type of response that develops. Apart from the type 17 T helper (Th17), Th22 and regulatory T (Treg) cells, the CD4^+^ T cells are divided majorly into two subsets: Th1 and Th2, according to their patterns of cytokine production. Th1 cells mainly secrete IFN-γ, IL-2, and IL-12 and other associated proinflammatory cytokines, while the Th2 cells secrete large amounts of IL-4, IL-5 and IL-13 to promote collagen synthesis by fibroblasts [43]. However, there is the possibility that Th1 may also contribute to fibrogenesis, as Th1 and Th2 cytokines can cross-regulate each other’s responses [43]. For instance, the Th1 IFN-γ cytokine may exacerbate fibrotic disease by downregulating the IL-13 decoy receptor (IL-13Rα2) [44], functioning as a soluble fusion protein that effectively inhibits IL-13 activity [45], blocking the initial collagen production during an inflammatory response [46,47], and ameliorating the progression of established fibrotic disease [48,49].

Furthermore, in principle, it is believed that the pathogenesis of fibrogenic and fibroprogressive diseases is importantly driven by the transforming growth factor-β1 (TGF-β1) [50], a cytokine that can be synthesized by diverse cells, including keratinocytes, fibroblasts, monocytes, macrophages, chondrocytes, platelets, epithelial cells and some T cells [43,51]. Nevertheless, clinical and functional assessment of the role of TGF-β1 in podoconiosis appears to disrupt the paradigm, hypothesizing that podoconiosis-susceptible individuals have low expression of TGF-β1 [42]. Implicating the possible fibrogenic role of the pathology-induced B cell infiltration in podoconiosis, a subset of B cells comprising IL-10-producing regulatory B cells, termed B10, has been recently identified in an analogous silica-induced lung fibrosis known as pneumoconiosis or silicosis [52]. B10 is silica-inducible and may suppress inflammation, while exacerbating fibrosis by inhibiting Th1 response, modulating the Th balance, promoting Treg induction and secreting IL-10 [52].

## 3. Immunological Role of Hookworm Co-Infection in Podoconiosis

**Figure 1:**
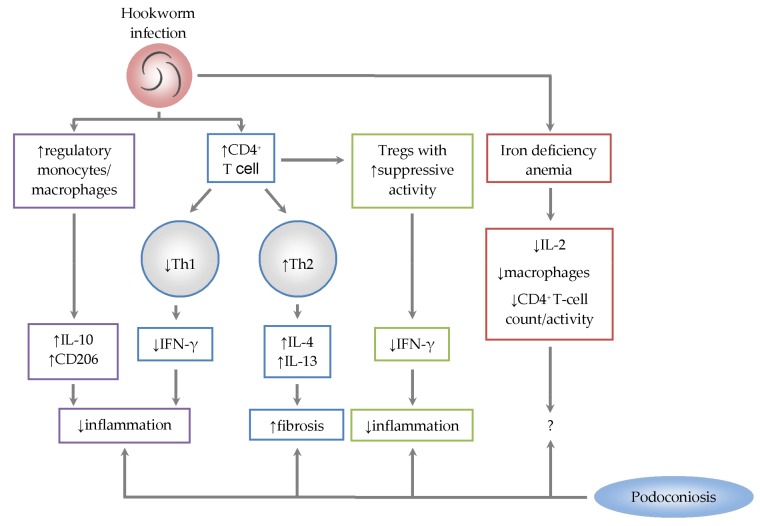
Possible immunological interrelation during podoconiosis and hookworm co-infection. Hookworm infection stimulates the activation of CD4^+^ T cells, induces downregulation and upregulation of the Th1 and Th2 responses respectively, and upregulates the suppressive activity of the regulatory T cells (Tregs) that reduce IFN-γ expression. Progressive retardation in IFN-γ levels with increasing worm burden and the induced increase in regulatory CD206^+^ and/or IL-10^+^ monocytes/macrophages may ameliorate inflammation but the upregulated Th2 (such as IL-4 and IL-13) response promotes fibrosis. Iron deficiency anemia caused by heavy hookworm burden may result in reduced IL-2 secretion, reduced number of macrophages and reduced CD4^+^ T-cell count or activity, but the impact of these outcomes in the pathology of podoconiosis appears elusive.

Modulation of the human immune responses by hookworms is an adaptation strategy to ensure prolonged survival within the host. *Ancylostoma* hookworms may modulate host cellular immune responses through multiple mechanisms, such as reduced mitogen-mediated lymphocyte proliferation, impaired antigen presentation/processing and relative reductions in CD4^+^ T-cell populations in the spleen and mesenteric lymph nodes [53]. Studies on experimental and natural human infections with hookworms have observed that the infection triggers strong Th2 cytokines (especially IL-4, IL-5, IL-9 and IL-13), regulatory IL-10 and TGF-β1 responses [54,55,56,57,58]. Release of the Th1 IFN-γ and IL-2 may also be induced [56]. However, progressive suppression of the IFN-γ response with increasing worm burden has been observed [54]. Similarly, the high frequency of circulating monocytes with a regulatory profile thatpromotes the down-modulation of the proinflammatory response was observed in *N. americanus*-infected individuals [59]. Infection with *N. americanus* may not affect the levels of IL-4 and arginase-1 (Arg-1) expression by the M2 macrophages, although it results inhigher numbers of CD206^+^CD23^+^IL-10^+^ monocytes [59]. Conversely, hookworm infection does not seem to affect the frequency of Tregs (CD4^+^CD25^hi^FOXP3^+^). However, suppressive activity of the Tregs differs between infected and uninfected individuals as the Tregs suppress production of the proinflammatory IFN-γ in infected individuals [60] (Figure 1). Arg-1 and CD206 (mannose receptor) are expressed markers of the tissue repair M2 macrophages [61]. CD206 majorly functions in M2 phagocytosis activity and resolution of inflammation, while Arg-1 may be strongly associated with the development of fibrosis [43,61] by mediating the conversion of arginine to polyamines and hydroxyproline, which directly contribute to ECM synthesis [62].

Additionally, percutaneous penetration by hookworm L3 larvae causes skin rashes and intensely itchy, erythematous, papulovesicular lesions localized to the site of entry. However, a creeping eruption known as cutaneous larva migrans is caused by the human skin-invading/resident zoonotic hookworm species [19,24,63]. The dermatitis occurs as a result of a strong, localized Th2 response characterized by an eosinophil-rich inflammatory infiltrate induced by the invasive larvae [64,65]. Therefore, this may possibly contribute partly to the skin damage or exacerbation of fibrosis in the mineral particle-induced elephantiasis.

Heavy hookworm infection causes IDA, which may adversely affect the immune system. Generally, studies have emphasized that IDA significantly impairs the integrity of both innate and cell-mediated immunity. For instance, the immune system requires iron for monocyte-macrophage differentiation [66]. Its deficiency may affect leukocyte phagocytic functions [67] and reduce neutrophil count and phagocytic activity [66]. Although the reduction in macrophage or monocyte count may reduce ECM deposition and ameliorate fibrosis [38], it has also been demonstrated that reduced monocyte-derived macrophages may orchestrate diffuse fibrotic development [68]. IDA may also cause reduced lymphocyte population [67]. Specifically, IDA was observed to cause significantly low CD4^+^ T-cell levels and decreased the ratio of mature T lymphocytes (CD4^+^:CD8^+^) [69]. The reductions were attributed to the decreased lymphocytic production of IL-2 [69] which serves as a T-cell growth factor and induces clonal T-cell proliferationin principle [51] (Figure 1). Conversely, a recent study observed an insignificant difference in CD4^+^ and CD4^+^:CD8^+^ levels between IDA and non-IDA human groups [70] and it has been suggested that IDA may cause functional defects of T cells rather than quantitative defects [66]. From these contrasting reports, it may be suggested that IDA causes functional and/or numerical defects in T cells (Figure 1), although this could possibly depend on some factors yet unknown. Nevertheless, high *Ancylostoma* hookworm burden has been found to be associated with severe IDA and concomitant depletion of CD4^+^ T cells in animal models [53], but the effect of CD4^+^ T-cell depletion on fibrogenesis appears controversial (Figure 1). While the depletion of CD4^+^ T cells may dampen fibroblast differentiation and subsequent ECM accumulation, thereby attenuating fibrosis [71], it has also been shown that reduced CD4^+^ T cells during human immunodeficiency virus and hepatitis C virus (HIV/HCV) co-infection may promote hepatic fibroprogression [72,73].

## 4. Conclusions

The involvement of concomitant hookworm infection in the development and progression of podoconiosis seems dynamic. Although co-occurrence with light, moderate or heavy hookworm infection has the potential to attenuate inflammatory responses, it may also contribute to fibrogenesis. Nevertheless, the possible impact of IDA-established hookworm infection in podoconiosis fibrogenesis and fibroprogression appears to still be inconclusive. Considering the high occurrence of hookworm infection and/or IDA among podoconiosis patients, deworming and iron-rich nutrient supplementation were recommended in addition to the basic podoconiosis treatment for patients living under resource-poor conditions to improve their well-being [28]. It is conceivable from the present review that the iron supplementation approach might not be applicable to patients, who are at either early or advanced clinical stages of the disease, as it may result inprovoked or exaggerated fibrotic responses. Podoconiosis-unaffected individuals, who are iron-deficient and living in at-risk areas, would be more eligible for iron supplementation. It is imperative to emphasize that iron deficiency can solely influence podoconiosis, but the fact that IDA-causal hookworms are predominant in podoconiosis patients makes it of more parasitological than mere nutritional or dietary importance. Finally, since the impact of hookworm co-infection in the pathology of podoconiosis is still not conclusively clear, we recommend diagnosing hookworm infections in podoconiosis-affected individuals, who respond poorly to the basic podoconiosis treatment, followed by proper deworming in applicable cases and preventive deworming of individuals living in co-endemic areas.
